# Napropamide

**DOI:** 10.1107/S1600536813017947

**Published:** 2013-07-06

**Authors:** Seonghwa Cho, Jineun Kim, Youngeun Jeon, Tae Ho Kim

**Affiliations:** a Department of Chemistry and Research Institute of Natural Sciences, Gyeongsang National University, Jinju 660-701, Republic of Korea

## Abstract

The title compound [systematic name: *N*,*N*-diethyl-2-(naphthalen-1-yl­oxy)propanamide], C_17_H_21_NO_2_, crystallizes with two independent mol­ecules in the asymmetric unit in which the dihedral angles between the naphthalene ring systems and the amide groups are 88.1 (9) and 88.7 (3)°. Four C—H⋯O hydrogen bonds stabilize the crystal structure.

## Related literature

For the herbicidal effects of the title compound, see: Freeman (1986 ▶). For information on the synthesis of the title compound, see: Gless (1986 ▶). For a related crystal structure, see: Au-Yeung *et al.* (2009 ▶).
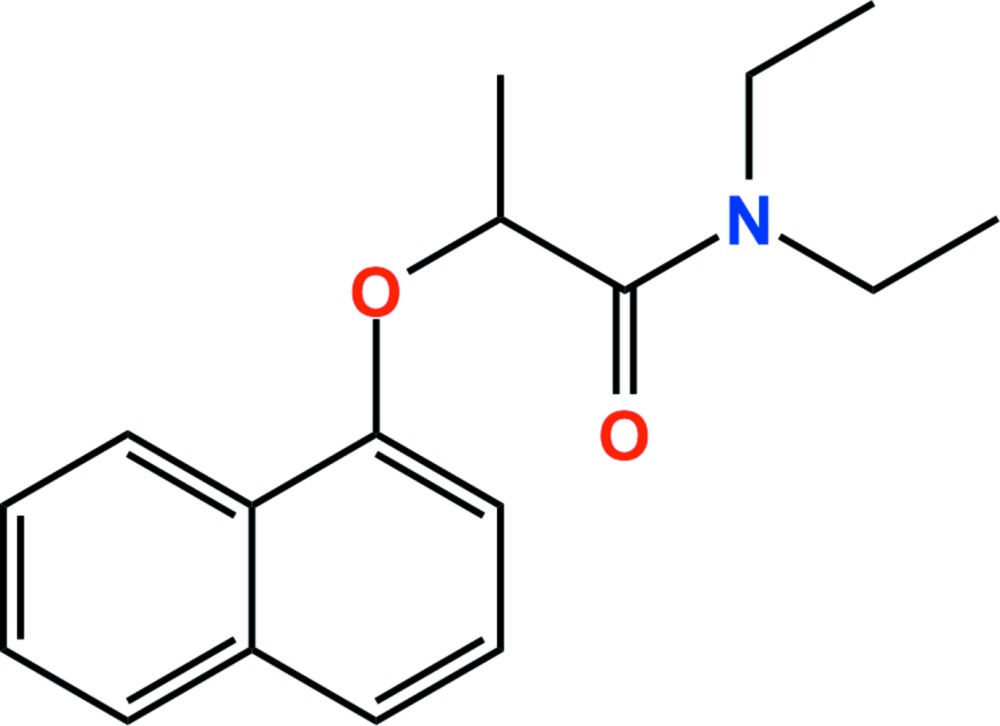


## Experimental

### 

#### Crystal data

C_17_H_21_NO_2_
*M* *_r_* = 271.35
Monoclinic, 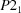

*a* = 9.8733 (3) Å
*b* = 10.7710 (4) Å
*c* = 14.1044 (5) Å
β = 97.943 (2)°
*V* = 1485.55 (9) Å^3^
*Z* = 4
Mo *K*α radiation
μ = 0.08 mm^−1^
*T* = 173 K
0.35 × 0.18 × 0.15 mm

#### Data collection

Bruker APEXII CCD diffractometer
Absorption correction: multi-scan (*SADABS*; Bruker, 2009 ▶) *T*_min_ = 0.973, *T*_max_ = 0.988
26573 measured reflections
3897 independent reflections
3297 reflections with *I* > 2σ(*I*)
*R*_int_ = 0.041

#### Refinement

*R*[*F* ^2^ > 2σ(*F* ^2^)] = 0.037
*wR*(*F* ^2^) = 0.083
*S* = 1.03
3897 reflections
367 parameters
1 restraint
H-atom parameters constrained
Δρ_max_ = 0.15 e Å^−3^
Δρ_min_ = −0.16 e Å^−3^

### 

Data collection: *APEX2* (Bruker, 2009 ▶); cell refinement: *SAINT* (Bruker, 2009 ▶); data reduction: *SAINT*; program(s) used to solve structure: *SHELXTL* (Sheldrick, 2008 ▶); program(s) used to refine structure: *SHELXTL*; molecular graphics: *SHELXTL*; software used to prepare material for publication: *SHELXTL*.

## Supplementary Material

Crystal structure: contains datablock(s) global, I. DOI: 10.1107/S1600536813017947/sj5338sup1.cif

Structure factors: contains datablock(s) I. DOI: 10.1107/S1600536813017947/sj5338Isup2.hkl

Supplementary material file. DOI: 10.1107/S1600536813017947/sj5338Isup3.cml

Additional supplementary materials:  crystallographic information; 3D view; checkCIF report

## Figures and Tables

**Table 1:** Hydrogen-bond geometry (Å, °)

*D*—H⋯*A*	*D*—H	H⋯*A*	*D*⋯*A*	*D*—H⋯*A*
C25—H25⋯O2	0.95	2.41	3.228 (3)	144
C6—H6⋯O2^i^	0.95	2.53	3.388 (3)	150
C7—H7⋯O4^ii^	0.95	2.60	3.481 (3)	154
C23—H23⋯O4^iii^	0.95	2.46	3.376 (3)	161

Symmetry codes: (i) 
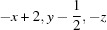
; (ii) 
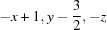
; (iii) 
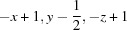
.

**Fig. 1.**
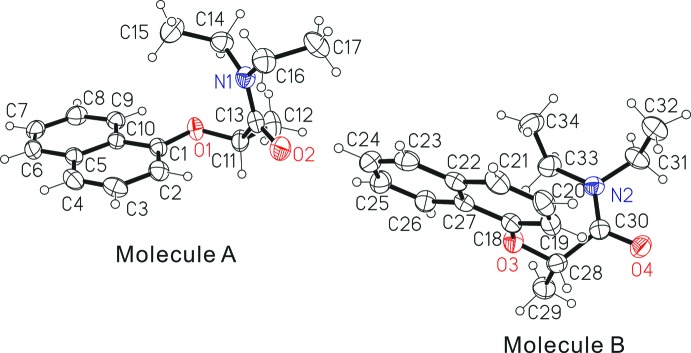
The asymmetric unit of the title compound with the atom numbering scheme. Displacement ellipsoids are drawn at the 50% probability level. Hydrogen atoms are represented by small spheres of arbitrary radius.

**Fig. 2.**
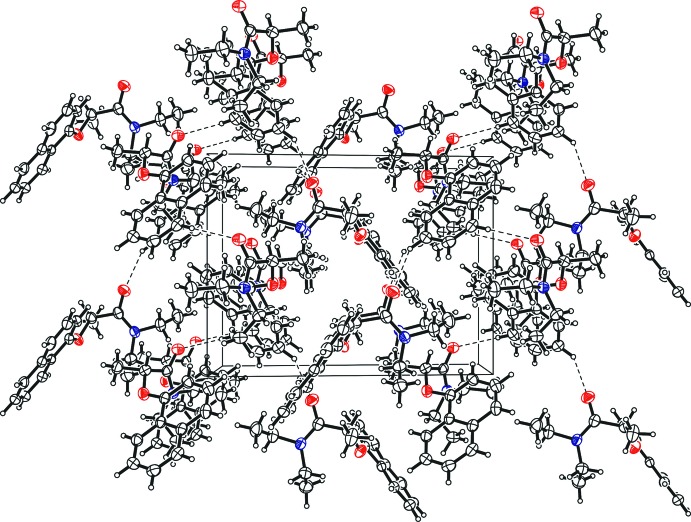
Crystal packing viewed along the a axis. The intermolecular C—H···O hydrogen bonds are shown as dashed lines.

## supplementary crystallographic information

### Comment

Napropamide (systematic name: *N*, *N*-diethyl-2-(1-naphthalenyloxy) propanamide) is a residual herbicide of the amide series. It is used as a control method for broadleaf grasses and annual grasses (Freeman, 1986). However its crystal structure has not yet been reported.

In the title compound (Scheme 1, Fig. 1), crystallizes with two independent molecules (Molecule A and Molecule B) in the asymmetric unit. The dihedral angles between the naphthalene ring systems and the amide groups are 88.1 (9) and 88.7 (3)° for Molecule A and Molecule B, respectively. C11 and C28 are chiral centres. All bond lengths and bond angles are normal and comparable to those observed in a similar crystal structure (Au-Yeung *et al.*, 2009). In the crystal structure (Fig. 2), four C—H···O hydrogen bonds are observed (Table 1). These intermolecular interactions may contribute to the stabilization of the packing.

### Experimental

The title compound was purchased from the Dr. Ehrenstorfer GmbH Company. Slow evaporation of a solution in CH_2_Cl_2_ gave single crystals suitable for X-ray analysis.

### Refinement

All H-atoms were positioned geometrically and refined using a riding model with d(C—H) = 0.95 Å, *U*_iso_ = 1.2*U*_eq_(C) for aromatic C—H, d(C—H) =1.00 Å, *U*_iso_ = 1.2*U*_eq_(C) for chiral C—H, d(C—H) = 0.99 Å, *U*_iso_ = 1.2*U*_eq_(C) for CH_2_ groups and d(C—H) = 0.98 Å, *U*_iso_ = 1.5*U*_eq_(C) for CH_3_ groups. In the absence of significant anomalous scattering effects, Friedel pairs were merged.

### Crystal data

**Table:**

C_17_H_21_NO_2_	*F*(000) = 584
*M**_r_* = 271.35	*D*_x_ = 1.213 Mg m^−^^3^
Monoclinic, *P*2_1_	Mo *K*α radiation, λ = 0.71073 Å
Hall symbol: P 2yb	Cell parameters from 9958 reflections
*a* = 9.8733 (3) Å	θ = 2.4–28.2°
*b* = 10.7710 (4) Å	µ = 0.08 mm^−^^1^
*c* = 14.1044 (5) Å	*T* = 173 K
β = 97.943 (2)°	Block, colourless
*V* = 1485.55 (9) Å^3^	0.35 × 0.18 × 0.15 mm
*Z* = 4	

### Data collection

**Table:**

Bruker APEXII CCD diffractometer	3897 independent reflections
Radiation source: fine-focus sealed tube	3297 reflections with *I* > 2σ(*I*)
Graphite monochromator	*R*_int_ = 0.041
φ and ω scans	θ_max_ = 28.3°, θ_min_ = 1.5°
Absorption correction: multi-scan (*SADABS*; Bruker, 2009)	*h* = −13→13
*T*_min_ = 0.973, *T*_max_ = 0.988	*k* = −12→14
26573 measured reflections	*l* = −18→18

### Refinement

**Table:**

Refinement on *F*^2^	Primary atom site location: structure-invariant direct methods
Least-squares matrix: full	Secondary atom site location: difference Fourier map
*R*[*F*^2^ > 2σ(*F*^2^)] = 0.037	Hydrogen site location: inferred from neighbouring sites
*wR*(*F*^2^) = 0.083	H-atom parameters constrained
*S* = 1.03	*w* = 1/[σ^2^(*F*_o_^2^) + (0.0333*P*)^2^ + 0.2525*P*] where *P* = (*F*_o_^2^ + 2*F*_c_^2^)/3
3897 reflections	(Δ/σ)_max_ < 0.001
367 parameters	Δρ_max_ = 0.15 e Å^−^^3^
1 restraint	Δρ_min_ = −0.16 e Å^−^^3^

### Special details

**Table:**

Geometry. All e.s.d.'s (except the e.s.d. in the dihedral angle between two l.s. planes) are estimated using the full covariance matrix. The cell e.s.d.'s are taken into account individually in the estimation of e.s.d.'s in distances, angles and torsion angles; correlations between e.s.d.'s in cell parameters are only used when they are defined by crystal symmetry. An approximate (isotropic) treatment of cell e.s.d.'s is used for estimating e.s.d.'s involving l.s. planes.
Refinement. Refinement of *F*^2^ against ALL reflections. The weighted *R*-factor *wR* and goodness of fit *S* are based on *F*^2^, conventional *R*-factors *R* are based on *F*, with *F* set to zero for negative *F*^2^. The threshold expression of *F*^2^ > σ(*F*^2^) is used only for calculating *R*-factors(gt) *etc*. and is not relevant to the choice of reflections for refinement. *R*-factors based on *F*^2^ are statistically about twice as large as those based on *F*, and *R*- factors based on ALL data will be even larger.

### Fractional atomic coordinates and isotropic or equivalent isotropic displacement parameters (Å^2^)

**Table:**

	*x*	*y*	*z*	*U*_iso_*/*U*_eq_	
O1	0.64959 (13)	0.16746 (14)	−0.03108 (9)	0.0340 (3)	
O2	0.66356 (17)	0.40562 (16)	0.14147 (11)	0.0447 (4)	
O3	0.33269 (15)	0.94175 (15)	0.27007 (9)	0.0365 (4)	
O4	0.10307 (16)	1.13370 (15)	0.37266 (11)	0.0410 (4)	
N1	0.67648 (18)	0.20374 (17)	0.18196 (11)	0.0343 (4)	
N2	0.07201 (18)	0.92770 (17)	0.35334 (11)	0.0328 (4)	
C1	0.78359 (19)	0.1750 (2)	−0.04740 (12)	0.0285 (4)	
C2	0.8753 (2)	0.2628 (2)	−0.00987 (13)	0.0335 (5)	
H2	0.8471	0.3282	0.0282	0.040*	
C3	1.0125 (2)	0.2560 (2)	−0.02794 (13)	0.0373 (5)	
H3	1.0764	0.3169	−0.0015	0.045*	
C4	1.0541 (2)	0.1635 (2)	−0.08256 (14)	0.0369 (5)	
H4	1.1471	0.1593	−0.0927	0.044*	
C5	0.9607 (2)	0.0737 (2)	−0.12436 (13)	0.0297 (4)	
C6	0.9996 (2)	−0.0220 (2)	−0.18405 (14)	0.0365 (5)	
H6	1.0921	−0.0277	−0.1952	0.044*	
C7	0.9068 (2)	−0.1059 (2)	−0.22572 (15)	0.0398 (5)	
H7	0.9348	−0.1690	−0.2659	0.048*	
C8	0.7705 (2)	−0.0996 (2)	−0.20960 (15)	0.0386 (5)	
H8	0.7064	−0.1584	−0.2391	0.046*	
C9	0.7284 (2)	−0.0093 (2)	−0.15167 (13)	0.0323 (4)	
H9	0.6355	−0.0062	−0.1412	0.039*	
C10	0.8220 (2)	0.0790 (2)	−0.10762 (12)	0.0273 (4)	
C11	0.5932 (2)	0.2710 (2)	0.01310 (14)	0.0361 (5)	
H11	0.6113	0.3469	−0.0241	0.043*	
C12	0.4395 (2)	0.2515 (3)	0.00062 (16)	0.0500 (7)	
H12A	0.4039	0.2498	−0.0678	0.075*	
H12B	0.3963	0.3196	0.0315	0.075*	
H12C	0.4189	0.1725	0.0300	0.075*	
C13	0.6490 (2)	0.2955 (2)	0.11836 (14)	0.0337 (5)	
C14	0.6488 (2)	0.0708 (2)	0.16476 (16)	0.0409 (5)	
H14A	0.5902	0.0607	0.1024	0.049*	
H14B	0.5976	0.0384	0.2151	0.049*	
C15	0.7780 (3)	−0.0051 (3)	0.16438 (17)	0.0506 (6)	
H15A	0.8216	0.0173	0.1084	0.076*	
H15B	0.7549	−0.0936	0.1616	0.076*	
H15C	0.8411	0.0119	0.2229	0.076*	
C16	0.7273 (2)	0.2395 (3)	0.28120 (14)	0.0436 (6)	
H16A	0.7921	0.3095	0.2806	0.052*	
H16B	0.7778	0.1687	0.3141	0.052*	
C17	0.6128 (3)	0.2773 (3)	0.33670 (16)	0.0516 (7)	
H17A	0.5676	0.3518	0.3077	0.077*	
H17B	0.6507	0.2948	0.4033	0.077*	
H17C	0.5461	0.2097	0.3350	0.077*	
C18	0.4283 (2)	0.9270 (2)	0.34998 (13)	0.0304 (4)	
C19	0.4394 (2)	1.0002 (2)	0.42959 (14)	0.0354 (5)	
H19	0.3775	1.0670	0.4334	0.043*	
C20	0.5439 (2)	0.9757 (2)	0.50642 (15)	0.0389 (5)	
H20	0.5523	1.0273	0.5616	0.047*	
C21	0.6324 (2)	0.8802 (2)	0.50295 (14)	0.0374 (5)	
H21	0.7016	0.8654	0.5555	0.045*	
C22	0.6222 (2)	0.8019 (2)	0.42105 (13)	0.0311 (4)	
C23	0.7123 (2)	0.7017 (2)	0.41399 (15)	0.0363 (5)	
H23	0.7823	0.6847	0.4656	0.044*	
C24	0.7002 (2)	0.6289 (2)	0.33414 (16)	0.0396 (5)	
H24	0.7624	0.5623	0.3305	0.048*	
C25	0.5972 (2)	0.6514 (2)	0.25768 (15)	0.0392 (5)	
H25	0.5897	0.6000	0.2025	0.047*	
C26	0.5073 (2)	0.7470 (2)	0.26198 (14)	0.0347 (5)	
H26	0.4367	0.7608	0.2101	0.042*	
C27	0.5183 (2)	0.8257 (2)	0.34330 (13)	0.0287 (4)	
C28	0.2398 (2)	1.0435 (2)	0.26756 (14)	0.0334 (5)	
H28	0.2949	1.1204	0.2838	0.040*	
C29	0.1716 (2)	1.0554 (2)	0.16425 (14)	0.0428 (6)	
H29A	0.2415	1.0701	0.1225	0.064*	
H29B	0.1074	1.1252	0.1590	0.064*	
H29C	0.1221	0.9787	0.1448	0.064*	
C30	0.1332 (2)	1.0349 (2)	0.33672 (13)	0.0308 (4)	
C31	−0.0305 (2)	0.9321 (3)	0.41976 (15)	0.0430 (6)	
H31A	−0.0973	0.8643	0.4034	0.052*	
H31B	−0.0807	1.0117	0.4108	0.052*	
C32	0.0295 (3)	0.9199 (3)	0.52358 (14)	0.0491 (6)	
H32A	0.0645	0.8354	0.5358	0.074*	
H32B	−0.0414	0.9368	0.5641	0.074*	
H32C	0.1044	0.9795	0.5384	0.074*	
C33	0.0882 (2)	0.8087 (2)	0.30606 (15)	0.0406 (5)	
H33A	0.1411	0.8222	0.2522	0.049*	
H33B	−0.0033	0.7774	0.2790	0.049*	
C34	0.1596 (3)	0.7109 (2)	0.37179 (18)	0.0500 (6)	
H34A	0.2475	0.7434	0.4027	0.075*	
H34B	0.1754	0.6368	0.3345	0.075*	
H34C	0.1023	0.6891	0.4207	0.075*	

### Atomic displacement parameters (Å^2^)

**Table:**

	*U*^11^	*U*^22^	*U*^33^	*U*^12^	*U*^13^	*U*^23^
O1	0.0290 (7)	0.0402 (9)	0.0339 (7)	0.0012 (7)	0.0085 (6)	−0.0103 (6)
O2	0.0540 (10)	0.0376 (10)	0.0447 (8)	0.0007 (8)	0.0144 (7)	−0.0084 (7)
O3	0.0372 (8)	0.0415 (9)	0.0296 (7)	0.0074 (7)	0.0008 (6)	−0.0029 (6)
O4	0.0492 (9)	0.0325 (9)	0.0410 (8)	0.0076 (8)	0.0058 (7)	0.0005 (7)
N1	0.0358 (9)	0.0380 (11)	0.0288 (8)	0.0021 (8)	0.0035 (7)	−0.0044 (8)
N2	0.0354 (10)	0.0329 (10)	0.0298 (8)	−0.0045 (8)	0.0035 (7)	0.0006 (8)
C1	0.0278 (10)	0.0350 (11)	0.0228 (8)	0.0022 (9)	0.0037 (7)	0.0007 (8)
C2	0.0393 (11)	0.0358 (12)	0.0255 (8)	−0.0017 (10)	0.0055 (8)	−0.0041 (8)
C3	0.0359 (11)	0.0457 (14)	0.0294 (10)	−0.0117 (10)	0.0016 (8)	0.0033 (10)
C4	0.0284 (10)	0.0504 (15)	0.0324 (10)	−0.0008 (10)	0.0059 (8)	0.0066 (10)
C5	0.0299 (10)	0.0344 (11)	0.0253 (9)	0.0048 (9)	0.0062 (7)	0.0053 (8)
C6	0.0374 (12)	0.0414 (13)	0.0327 (10)	0.0111 (11)	0.0119 (8)	0.0079 (9)
C7	0.0579 (15)	0.0311 (12)	0.0330 (10)	0.0103 (11)	0.0158 (10)	0.0015 (9)
C8	0.0498 (13)	0.0315 (12)	0.0342 (10)	−0.0028 (11)	0.0051 (9)	−0.0029 (9)
C9	0.0350 (11)	0.0326 (11)	0.0296 (9)	−0.0004 (10)	0.0055 (8)	0.0010 (9)
C10	0.0300 (10)	0.0307 (11)	0.0216 (8)	0.0031 (9)	0.0048 (7)	0.0037 (8)
C11	0.0370 (11)	0.0389 (13)	0.0333 (10)	0.0089 (10)	0.0079 (8)	−0.0023 (9)
C12	0.0352 (12)	0.0755 (19)	0.0383 (11)	0.0150 (13)	0.0021 (9)	−0.0107 (12)
C13	0.0287 (10)	0.0413 (13)	0.0325 (10)	0.0028 (10)	0.0095 (8)	−0.0055 (9)
C14	0.0443 (13)	0.0376 (13)	0.0410 (11)	−0.0058 (11)	0.0063 (10)	0.0001 (10)
C15	0.0575 (15)	0.0432 (15)	0.0491 (13)	0.0111 (13)	0.0002 (11)	−0.0011 (12)
C16	0.0463 (13)	0.0534 (15)	0.0297 (9)	0.0013 (12)	0.0007 (9)	−0.0041 (10)
C17	0.0572 (15)	0.0623 (18)	0.0384 (11)	−0.0106 (14)	0.0177 (10)	−0.0067 (12)
C18	0.0275 (10)	0.0353 (11)	0.0292 (9)	−0.0017 (9)	0.0061 (7)	0.0024 (9)
C19	0.0343 (11)	0.0387 (13)	0.0349 (10)	−0.0026 (10)	0.0105 (8)	−0.0051 (10)
C20	0.0380 (12)	0.0475 (14)	0.0318 (10)	−0.0073 (11)	0.0073 (9)	−0.0090 (10)
C21	0.0325 (11)	0.0506 (14)	0.0285 (10)	−0.0045 (11)	0.0021 (8)	0.0010 (9)
C22	0.0275 (10)	0.0363 (12)	0.0306 (9)	−0.0052 (9)	0.0079 (7)	0.0042 (9)
C23	0.0330 (11)	0.0400 (13)	0.0351 (10)	−0.0011 (10)	0.0021 (8)	0.0102 (10)
C24	0.0398 (12)	0.0334 (12)	0.0468 (12)	0.0043 (10)	0.0097 (10)	0.0057 (10)
C25	0.0458 (13)	0.0350 (13)	0.0372 (10)	0.0017 (11)	0.0067 (9)	−0.0042 (10)
C26	0.0361 (11)	0.0368 (12)	0.0304 (9)	0.0021 (10)	0.0022 (8)	0.0002 (9)
C27	0.0281 (10)	0.0324 (11)	0.0267 (9)	−0.0031 (9)	0.0072 (7)	0.0031 (8)
C28	0.0338 (11)	0.0313 (12)	0.0353 (10)	0.0006 (9)	0.0056 (8)	0.0034 (9)
C29	0.0485 (14)	0.0482 (15)	0.0316 (10)	0.0072 (12)	0.0047 (9)	0.0074 (10)
C30	0.0298 (11)	0.0340 (12)	0.0273 (9)	0.0037 (9)	−0.0010 (8)	0.0033 (9)
C31	0.0321 (12)	0.0571 (16)	0.0409 (11)	−0.0066 (11)	0.0089 (9)	0.0061 (11)
C32	0.0525 (16)	0.0614 (17)	0.0342 (11)	−0.0045 (14)	0.0092 (10)	0.0026 (12)
C33	0.0489 (13)	0.0338 (12)	0.0376 (11)	−0.0094 (11)	0.0008 (9)	−0.0059 (10)
C34	0.0729 (17)	0.0295 (12)	0.0474 (13)	−0.0043 (12)	0.0074 (12)	−0.0008 (11)

### Geometric parameters (Å, º)

**Table:**

O1—C1	1.376 (2)	C16—C17	1.517 (3)
O1—C11	1.428 (3)	C16—H16A	0.9900
O2—C13	1.233 (3)	C16—H16B	0.9900
O3—C18	1.376 (2)	C17—H17A	0.9800
O3—C28	1.426 (3)	C17—H17B	0.9800
O4—C30	1.233 (3)	C17—H17C	0.9800
N1—C13	1.337 (3)	C18—C19	1.364 (3)
N1—C16	1.471 (3)	C18—C27	1.419 (3)
N1—C14	1.472 (3)	C19—C20	1.414 (3)
N2—C30	1.339 (3)	C19—H19	0.9500
N2—C33	1.464 (3)	C20—C21	1.355 (3)
N2—C31	1.472 (3)	C20—H20	0.9500
C1—C2	1.364 (3)	C21—C22	1.422 (3)
C1—C10	1.423 (3)	C21—H21	0.9500
C2—C3	1.414 (3)	C22—C23	1.411 (3)
C2—H2	0.9500	C22—C27	1.418 (3)
C3—C4	1.357 (3)	C23—C24	1.364 (3)
C3—H3	0.9500	C23—H23	0.9500
C4—C5	1.408 (3)	C24—C25	1.398 (3)
C4—H4	0.9500	C24—H24	0.9500
C5—C6	1.417 (3)	C25—C26	1.366 (3)
C5—C10	1.423 (3)	C25—H25	0.9500
C6—C7	1.361 (3)	C26—C27	1.418 (3)
C6—H6	0.9500	C26—H26	0.9500
C7—C8	1.397 (3)	C28—C29	1.524 (3)
C7—H7	0.9500	C28—C30	1.534 (3)
C8—C9	1.371 (3)	C28—H28	1.0000
C8—H8	0.9500	C29—H29A	0.9800
C9—C10	1.409 (3)	C29—H29B	0.9800
C9—H9	0.9500	C29—H29C	0.9800
C11—C12	1.519 (3)	C31—C32	1.507 (3)
C11—C13	1.533 (3)	C31—H31A	0.9900
C11—H11	1.0000	C31—H31B	0.9900
C12—H12A	0.9800	C32—H32A	0.9800
C12—H12B	0.9800	C32—H32B	0.9800
C12—H12C	0.9800	C32—H32C	0.9800
C14—C15	1.516 (3)	C33—C34	1.512 (3)
C14—H14A	0.9900	C33—H33A	0.9900
C14—H14B	0.9900	C33—H33B	0.9900
C15—H15A	0.9800	C34—H34A	0.9800
C15—H15B	0.9800	C34—H34B	0.9800
C15—H15C	0.9800	C34—H34C	0.9800
			
C1—O1—C11	117.92 (16)	H17A—C17—H17B	109.5
C18—O3—C28	118.37 (16)	C16—C17—H17C	109.5
C13—N1—C16	117.11 (19)	H17A—C17—H17C	109.5
C13—N1—C14	126.36 (17)	H17B—C17—H17C	109.5
C16—N1—C14	116.26 (18)	C19—C18—O3	125.1 (2)
C30—N2—C33	126.51 (17)	C19—C18—C27	121.31 (18)
C30—N2—C31	116.8 (2)	O3—C18—C27	113.55 (17)
C33—N2—C31	116.49 (19)	C18—C19—C20	119.3 (2)
C2—C1—O1	125.30 (18)	C18—C19—H19	120.3
C2—C1—C10	121.22 (17)	C20—C19—H19	120.3
O1—C1—C10	113.47 (17)	C21—C20—C19	121.3 (2)
C1—C2—C3	119.63 (19)	C21—C20—H20	119.3
C1—C2—H2	120.2	C19—C20—H20	119.3
C3—C2—H2	120.2	C20—C21—C22	120.38 (19)
C4—C3—C2	120.9 (2)	C20—C21—H21	119.8
C4—C3—H3	119.6	C22—C21—H21	119.8
C2—C3—H3	119.6	C23—C22—C27	118.64 (19)
C3—C4—C5	120.68 (19)	C23—C22—C21	122.44 (19)
C3—C4—H4	119.7	C27—C22—C21	118.9 (2)
C5—C4—H4	119.7	C24—C23—C22	121.0 (2)
C4—C5—C6	122.21 (19)	C24—C23—H23	119.5
C4—C5—C10	119.48 (18)	C22—C23—H23	119.5
C6—C5—C10	118.30 (19)	C23—C24—C25	120.6 (2)
C7—C6—C5	121.2 (2)	C23—C24—H24	119.7
C7—C6—H6	119.4	C25—C24—H24	119.7
C5—C6—H6	119.4	C26—C25—C24	120.3 (2)
C6—C7—C8	120.2 (2)	C26—C25—H25	119.9
C6—C7—H7	119.9	C24—C25—H25	119.9
C8—C7—H7	119.9	C25—C26—C27	120.57 (19)
C9—C8—C7	120.7 (2)	C25—C26—H26	119.7
C9—C8—H8	119.7	C27—C26—H26	119.7
C7—C8—H8	119.7	C22—C27—C26	118.95 (19)
C8—C9—C10	120.5 (2)	C22—C27—C18	118.71 (18)
C8—C9—H9	119.8	C26—C27—C18	122.33 (17)
C10—C9—H9	119.8	O3—C28—C29	106.63 (17)
C9—C10—C5	119.18 (18)	O3—C28—C30	115.67 (17)
C9—C10—C1	122.77 (17)	C29—C28—C30	111.22 (17)
C5—C10—C1	118.05 (18)	O3—C28—H28	107.7
O1—C11—C12	106.54 (18)	C29—C28—H28	107.7
O1—C11—C13	116.58 (17)	C30—C28—H28	107.7
C12—C11—C13	110.83 (16)	C28—C29—H29A	109.5
O1—C11—H11	107.5	C28—C29—H29B	109.5
C12—C11—H11	107.5	H29A—C29—H29B	109.5
C13—C11—H11	107.5	C28—C29—H29C	109.5
C11—C12—H12A	109.5	H29A—C29—H29C	109.5
C11—C12—H12B	109.5	H29B—C29—H29C	109.5
H12A—C12—H12B	109.5	O4—C30—N2	122.14 (19)
C11—C12—H12C	109.5	O4—C30—C28	115.79 (19)
H12A—C12—H12C	109.5	N2—C30—C28	121.99 (18)
H12B—C12—H12C	109.5	N2—C31—C32	113.77 (18)
O2—C13—N1	121.89 (19)	N2—C31—H31A	108.8
O2—C13—C11	115.7 (2)	C32—C31—H31A	108.8
N1—C13—C11	122.3 (2)	N2—C31—H31B	108.8
N1—C14—C15	112.8 (2)	C32—C31—H31B	108.8
N1—C14—H14A	109.0	H31A—C31—H31B	107.7
C15—C14—H14A	109.0	C31—C32—H32A	109.5
N1—C14—H14B	109.0	C31—C32—H32B	109.5
C15—C14—H14B	109.0	H32A—C32—H32B	109.5
H14A—C14—H14B	107.8	C31—C32—H32C	109.5
C14—C15—H15A	109.5	H32A—C32—H32C	109.5
C14—C15—H15B	109.5	H32B—C32—H32C	109.5
H15A—C15—H15B	109.5	N2—C33—C34	113.70 (18)
C14—C15—H15C	109.5	N2—C33—H33A	108.8
H15A—C15—H15C	109.5	C34—C33—H33A	108.8
H15B—C15—H15C	109.5	N2—C33—H33B	108.8
N1—C16—C17	112.36 (19)	C34—C33—H33B	108.8
N1—C16—H16A	109.1	H33A—C33—H33B	107.7
C17—C16—H16A	109.1	C33—C34—H34A	109.5
N1—C16—H16B	109.1	C33—C34—H34B	109.5
C17—C16—H16B	109.1	H34A—C34—H34B	109.5
H16A—C16—H16B	107.9	C33—C34—H34C	109.5
C16—C17—H17A	109.5	H34A—C34—H34C	109.5
C16—C17—H17B	109.5	H34B—C34—H34C	109.5
			
C11—O1—C1—C2	12.6 (3)	C28—O3—C18—C19	2.2 (3)
C11—O1—C1—C10	−168.54 (16)	C28—O3—C18—C27	−177.86 (17)
O1—C1—C2—C3	176.36 (18)	O3—C18—C19—C20	−179.1 (2)
C10—C1—C2—C3	−2.4 (3)	C27—C18—C19—C20	0.9 (3)
C1—C2—C3—C4	0.3 (3)	C18—C19—C20—C21	−0.7 (3)
C2—C3—C4—C5	1.4 (3)	C19—C20—C21—C22	0.2 (3)
C3—C4—C5—C6	178.1 (2)	C20—C21—C22—C23	179.8 (2)
C3—C4—C5—C10	−1.1 (3)	C20—C21—C22—C27	0.1 (3)
C4—C5—C6—C7	−178.3 (2)	C27—C22—C23—C24	0.1 (3)
C10—C5—C6—C7	1.0 (3)	C21—C22—C23—C24	−179.6 (2)
C5—C6—C7—C8	−0.5 (3)	C22—C23—C24—C25	−0.6 (3)
C6—C7—C8—C9	−0.1 (3)	C23—C24—C25—C26	0.1 (3)
C7—C8—C9—C10	0.2 (3)	C24—C25—C26—C27	0.9 (3)
C8—C9—C10—C5	0.3 (3)	C23—C22—C27—C26	0.9 (3)
C8—C9—C10—C1	179.57 (19)	C21—C22—C27—C26	−179.40 (19)
C4—C5—C10—C9	178.36 (18)	C23—C22—C27—C18	−179.53 (18)
C6—C5—C10—C9	−0.9 (3)	C21—C22—C27—C18	0.2 (3)
C4—C5—C10—C1	−0.9 (3)	C25—C26—C27—C22	−1.4 (3)
C6—C5—C10—C1	179.84 (17)	C25—C26—C27—C18	179.0 (2)
C2—C1—C10—C9	−176.56 (18)	C19—C18—C27—C22	−0.7 (3)
O1—C1—C10—C9	4.5 (3)	O3—C18—C27—C22	179.40 (17)
C2—C1—C10—C5	2.7 (3)	C19—C18—C27—C26	178.9 (2)
O1—C1—C10—C5	−176.23 (16)	O3—C18—C27—C26	−1.1 (3)
C1—O1—C11—C12	166.67 (16)	C18—O3—C28—C29	166.45 (18)
C1—O1—C11—C13	−69.0 (2)	C18—O3—C28—C30	−69.3 (2)
C16—N1—C13—O2	−1.0 (3)	C33—N2—C30—O4	172.35 (19)
C14—N1—C13—O2	172.9 (2)	C31—N2—C30—O4	−2.1 (3)
C16—N1—C13—C11	−178.96 (18)	C33—N2—C30—C28	−4.3 (3)
C14—N1—C13—C11	−5.1 (3)	C31—N2—C30—C28	−178.82 (17)
O1—C11—C13—O2	141.9 (2)	O3—C28—C30—O4	143.88 (18)
C12—C11—C13—O2	−96.0 (2)	C29—C28—C30—O4	−94.3 (2)
O1—C11—C13—N1	−39.9 (3)	O3—C28—C30—N2	−39.2 (3)
C12—C11—C13—N1	82.1 (3)	C29—C28—C30—N2	82.6 (2)
C13—N1—C14—C15	110.4 (2)	C30—N2—C31—C32	−85.6 (3)
C16—N1—C14—C15	−75.7 (3)	C33—N2—C31—C32	99.4 (3)
C13—N1—C16—C17	81.5 (3)	C30—N2—C33—C34	112.8 (2)
C14—N1—C16—C17	−93.0 (3)	C31—N2—C33—C34	−72.7 (3)

### Hydrogen-bond geometry (Å, º)

**Table:**

*D*—H···*A*	*D*—H	H···*A*	*D*···*A*	*D*—H···*A*
C25—H25···O2	0.95	2.41	3.228 (3)	144
C6—H6···O2^i^	0.95	2.53	3.388 (3)	150
C7—H7···O4^ii^	0.95	2.60	3.481 (3)	154
C23—H23···O4^iii^	0.95	2.46	3.376 (3)	161

Symmetry codes: (i) −*x*+2, *y*−1/2, −*z*; (ii) −*x*+1, *y*−3/2, −*z*; (iii) −*x*+1, *y*−1/2, −*z*+1.
